# The Multi-Omics Analysis Revealed a Metabolic Regulatory System of Cecum in Rabbit with Diarrhea

**DOI:** 10.3390/ani12091194

**Published:** 2022-05-06

**Authors:** Jie Wang, Kaisen Zhao, Zhe Kang, Meigui Wang, Yang Chen, Huimei Fan, Siqi Xia, Songjia Lai

**Affiliations:** 1 College of Animal Science and Technology, Sichuan Agricultural University, Chengdu 611130, China; wjie68@163.com (J.W.); zhaofl0303@163.com (K.Z.); kz516837559@163.com (Z.K.); wmg1987797495@163.com (M.W.); chenyi154121@163.com (Y.C.); fanhuimei1998@163.com (H.F.); xiasiqi2020@163.com (S.X.); 2 Farm Animal Genetic Resources Exploration and Innovation Key Laboratory of Sichuan Province, Chengdu Campus, Sichuan Agricultural University, Chengdu 611130, China

**Keywords:** rabbit, cecum, diarrhea, microorganism, transcriptome, untargeted metabolomics

## Abstract

**Simple Summary:**

In July 2020, China completely banned the addition of antibiotics to feed, which had a huge impact on animal husbandry, such as the increased incidence of some intestinal diseases. In this study, the pathogenesis of diarrhea in rabbits fed with antibiotic-free diet was studied by multi-omics analysis. The study found that the relative abundances of Firmicutes and Proteobacteria in the cecum of diarrhea-afflicted rabbits changed significantly. Most of the differential expression genes identified were related to cecum inflammation and immune response, and a total of 652 differential metabolites were identified, mainly involved in inflammation-related metabolic pathways such as bile secretion, folic acid resistance, and tryptophan metabolism. In addition, Fournierella was positively correlated with myricetin and ursolic acid; thus, it might further cause bile secretion and tryptophan metabolism disorders, aggravate intestinal inflammation, change intestinal permeability, reduce host immunity, and lead to diarrhea in rabbits.

**Abstract:**

With the comprehensive prohibition of antibiotics in the feed industry in China, the incidence of diarrhea in rabbits increased, such as loss of appetite, vomiting, and excretion of atheromatous feces. In order to explore the pathological and the molecular mechanisms of the diarrhea in the rabbitry fed with antibiotic-free diet, we used microbial metagenomics, transcriptome, and non-targeted metabolomics sequencing. The results showed that the Firmicutes level was significantly decreased (*p* < 0.001) and the Proteobacteria level was significantly increased (*p* < 0.05). The functional enrichment of cecum revealed that most differentially expressed genes (DEGs) were expressed in immune, inflammatory, and metabolic processes. The enrichment of the cecal fecal metabolites focused on the bile secretion, antifolate resistance, and tryptophan metabolism pathways, which are mainly associated with inflammation. The results of correlation analysis showed that Fournierella was positively correlated with myricetin, ursolic acid, and furtherly might cause bile secretion and tryptophan metabolism disorder, aggravate intestinal inflammation, change intestinal permeability, and reduce host immunity, leading to diarrhea in rabbits. This study provides a theoretical basis for illustrating the reason for diarrhea and developing new feeds for the health of rabbits.

## 1. Introduction

Antibiotics have played an important role in animal feed since their discovery [1]. Antibiotics in animal feed could maintain animal intestinal health and reduce animal mortality [2,3]. However, overuse of antibiotics not only leads to drug resistance in animals, but also affects consumer health in animal products [4,5]. After the African swine fever, consumers have paid more attention to the abuse of antibiotics in feed. July 2020, China completely prohibited the addition of antibiotics in feed. Subsequently, a lot of rabbits developed diarrhea and loss of appetite, vomiting, and excretion of atheromatous feces [6], which caused reduced feed utilization, low immunity, reduced performance, and even death [7,8], which seriously affects the efficiency of rabbit production.

For animals, the gut is a multifunctional organ that carries microbes that interact with host nutrition, metabolism, and immune function [9,10,11]. The intestinal structure of rabbits is quite different from that of domestic animals such as pigs and chickens [12]. The cecum plays an important role in the digestive and immune functions of rabbits. Cecum microorganisms can secrete various digestive enzymes to improve the digestive ability, and also synthesis of mycoprotein by nitrogen-containing, which could be used again by rabbits [13,14,15]. If the balance of microorganisms is imbalanced, it will not only cause inflammation and oxidative stress, but also reduce the metabolites of microorganisms such as acetic acid and butyric acid, and many metabolites produced by microorganisms that have inhibitory effects on the growth and reproduction of pathogenic bacteria [16,17]. Furthermore, intestinal microbes also have the ability to activate macrophages, promote cytokine-mediated secretion, and enhance host disease resistance [9].

With the updating of biotechnology, multi-omics combined analysis can be verified and interpreted from different levels, which is more conducive to reveal the complex regulation mechanisms of animal growth and development [18,19,20]. Diarrhea in rabbits might cause intestinal flora disorder, change intestinal permeability, and then cause intestinal inflammation and metabolic dysfunction [21].Some studies, through multi-omit techniques discovery, found that intestinal microorganisms are involved in host nutrient uptake, transport, and metabolic process [22,23]. However, there are few studies on the multi-omics characteristics and differentially expressed genes of gut microbes and metabolites in rabbits with diarrhea. Thus, the aim of this study was to explore the metabolites related to diarrhea by exploring the differential microorganisms in cecum, and identify the differential immune factors, and establishment of a metabolic regulatory network in diarrhea with feeding of antibiotic-free diet. This research might provide a theoretical basis for further understanding the pathogenesis of diarrhea in rabbits and the preparation of new feeds with health effects.

## 2. Materials and Methods

### 2.1. Ethics Statement

This study was approved and conducted in accordance with the ethical standards of the Institutional Animal Care and Use Committee of the College of Animal Science and Technology, Sichuan Agricultural University, Sichuan, 611130, China.

### 2.2. Animals and Feeding Conditions

The study was carried out in a rabbitry in Leibo County, Sichuan Province. All rabbits were raised under the conventional feeding conditions in the rabbitry. Each rabbit was raised in a clean rabbit cage (600 × 600 × 500 mm), with free drinking water, and fed local commercial rabbit feed. In July 2020, the rabbitry used the antibiotic-free diet based on the national regulation. Subsequently, the rabbits showed typical diarrhea symptoms, such as decreased appetite, thin feces, and mixed blood and jelly-like mucus in feces.

### 2.3. Sample Collection

Twelve 40-day-old Hyplus female rabbits with similar body weight and no kinship within three generations were selected, including six rabbits with typical diarrhea symptoms (Dia_Ce) and six healthy rabbits (Con_Ce). After fasting for 24 h, the rabbits were slaughtered with conventional methods. Then, the cecum and its contents were collected immediately. Six microbial samples, transcriptome samples, and metabolome samples were collected separately from each rabbit, and all samples were stored in liquid nitrogen until they were sent to Novogene (Beijing) for sequencing.

### 2.4. Pathological Analysis

In this study, six rabbits with typical diarrhea symptoms and six normal rabbits were selected for observation of cecum morphology. After dehydration and paraffin embedding, the cecum tissue samples were sliced by RM2235 microtome (Leica, Germany). After xylene dewaxing, they were water washed for 20 min and stained with hematoxylin and eosin (HE). The histopathological features, using CX22 microscope (OLMPUS, Japan), were observed and photographed using Leica microscopic imaging system (DM1000, Leica, Germany).

### 2.5. DNA Extraction, 16S rRNA Sequencing, and Sequence Analysis

Total genome DNA from cecum feces was extracted using CTAB/SDS method [24]. Construction of library and sequencing was performed according to conventional methods. Sequence analysis was performed using UPARSE [25]. Sequences with similarity ≥97% were assigned to the same OTUs, and the chimeric sequences were determined by UCHIME [26]. Then, mothur method and SILVA SSUrRNA database were used to annotate the species classification information of each OTUs representative sequence [27,28]. Finally, the data of each sample were homogenized and analyzed by bioinformatics.

Alpha diversity is applied in analyzing complexity of species diversity for a sample through indices, including Chao1, Shannon, and Simpson et al.; all of these indices in our samples were calculated with QIIME (Version1.9.1, Novogene, Beijing, China) and displayed with R software (Version 2.15.3, Novogene, Beijing, China). We used a Venn map to identify the number of unique and common OTUs in different groups. LEfSe software was used for linear discriminant analysis (LDA) and effect size (LEfSe) analysis to identify different bacterial groups in different groups, and high-dimensional biomarkers were found. The screening value of LDA score was set as 4 by default to determine the most identifiable bacterial group.

### 2.6. Transcriptome Sequencing and Differentially Expressed Genes Analysis

RNA extraction, library construction, and sequencing were performed by Novogene (Beijing). To ensure the quality and reliability of data analysis, we needed to filter the original data (mainly including the removal of low-quality reads, reads containing N (N means undetermined base information), and reads with adapters) and calculate the contents of Q20, Q30, and GC in the clean data. Then, HISAT2 was used to compare the paired clean reads with the reference genome of rabbits to obtain the localization information of reads on the reference genome [29]. Differentially expressed genes (DEGs) were analyzed using DESeq2 [30] and edgeR [31] software. The *p*-values were adjusted using the Benjamini and Hochberg method. Corrected *p*-value of 0.05 and absolute foldchange of 2 were set as the threshold for significantly differential expression. ClusterProfile software was used for GO functional enrichment analysis and KEGG pathway enrichment analysis of differential gene sets. A *p*-value < 0.05 was used as the threshold of significant enrichment for both GO and KEGG enrichment analysis. 

### 2.7. UHPLC-MS/MS-Based Non-Targeted Metabolomics Analysis

The metabolites in cecum contents were extracted with 80% methanol aqueous solution. Each sample was detected by LC–MS with 20 μL for quality control (QC). UHPLC–MS/MS analysis was completed in Novogene (Beijing). The raw data files generated by UHPLC–MS/MS were processed using the Compound Discoverer 3.1 (CD3.1, Thermo Fisher, Wilmington, DE, USA) to perform peak alignment, peak picking, and quantitation for each metabolite [32].

Principal component analysis (PCA) and partial least squares discriminant analysis (PLS-DA) [33] were performed at metaX [34]. We applied univariate analysis (*t*-test) to calculate the statistical significance (*p*-value). The metabolites with VIP (variable importance in the projection) > 1 and *p*-value < 0.05 and FC (fold change) ≥ 2 or FC ≤ 0.5 were considered to be differential metabolites [35,36,37]. Volcano plots were used to filter metabolites of interest which were based on log2 (fold change) and −log10 (*p*-value) of metabolites. Finally, the functions of these metabolites and metabolic pathways were studied using the KEGG database; when *p*-value of metabolic pathway < 0.05, metabolic pathway was considered as statistically significant enrichment.

### 2.8. Association Analysis

Pearson correlation analysis was employed to measure the correlation between microbial community diversity and metabolites in cecum feces samples, and the correlation coefficient (r) was (−1, 1). Two experimental factors were negatively correlated when r < 0; when r > 0, two experiment factors were positively correlated. If r = 0, there was no correlation between two experiment factors. All the obtained differential genes and differential metabolites were mapped to the KEGG pathway database to obtain their common pathway information.

### 2.9. Statistical Analysis

Statistical analyses were performed using SPSS 27.0 software (IBM, Chicago, IL, USA), and the significant difference analysis between the two groups were performed with *t*-test. A *p*-value < 0.05 showed significant difference, and *p*-value < 0.01 showed extremely significant difference.

## 3. Results

### 3.1. Pathological Features

**Figure 1:**
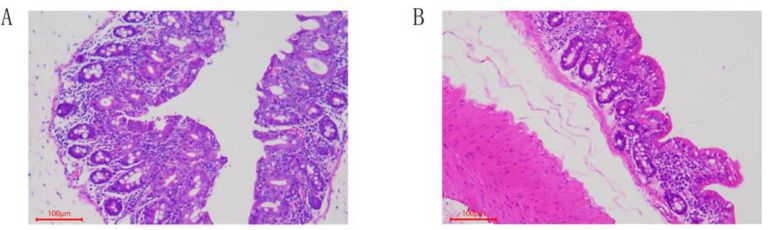
Pathological observation of cecum tissue in rabbits by microscope (HE-staining, 100×). (**A**) Dia_Ce. (**B**) Con_Ce.

The HE-staining result of the cecum tissue sections is shown in Figure 1. The cecum mucosal layer of the Dia_Ce group was hyperemia, the cells fell off locally in the epithelium, and the lymphocytes increased in the lamina propria. Conversely, the cecum physiological structure of the Con_Ce group was complete, and the cells were arranged neatly, without obvious histopathological damage.

### 3.2. The Diversity of Fecal Microflora

**Figure 2:**
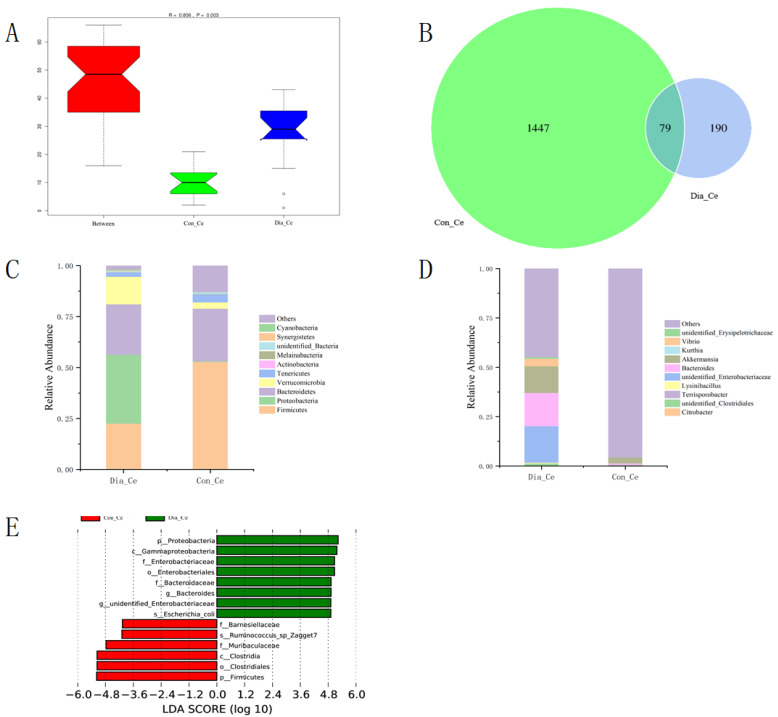
Comparison of fecal flora in cecum between Dia_Ce and Con_Ce. (**A**) ANOSIM analysis: R > 0, significant difference in two groups. (**B**) The number of unique and common OTUs between the two groups shown in the Venn diagram. (**C**) Relative abundance histogram of species at phylum level. (**D**) Relative abundance histogram of species at genus level. (**E**) LDA score of LEfSe–PICRUSt.

A total of 79,069 clean reads were obtained, with an average of 1098 reads per sample (Table S1). Then we used ANOSIM (Figure 2A) to visualize the differences between different groups. There were significant differences in microbial composition between the two groups. The Chao1 index, Shannon index, and Simpson index were significantly decreased in Dia_Ce (Table 1). Unique OUT in Dia_Ce was significantly lower than that in Con_Ce (Figure 2B).

The dominant groups of cecum bacteria were basically the same, which were Firmicutes, Bacteroidetes, and Proteobacteria (accounting for 78.89~81.05% of the total bacteria) (Table S2), but its abundance showed difference between the two groups. At the phylum level, the relative abundance of Firmicutes decreased significantly (*p* < 0.01), while the relative abundance of Proteobacteria increased significantly (*p* < 0.05). In addition, the relative abundance of Verrucomicrobia, Synergistetes, and Cyanobacteria increased in the Dia_Ce group, while the relative abundance of Actinobacteria and Bacteroidetes decreased (Figure 2C). At the order level, the microbial flora in Dia_Ce was mainly Enterobacteriales (26.47%), while the microbial flora in Con_Ce were mainly Clostridiales and Bacteroidia, and their relative abundances reached 49.30% and 25.71%, respectively (Table 2). The microbial composition of the two groups showed similar differences at genus level (Figure 2D). Then, the differences of bacterial groups were analyzed by LEfSe, and the characteristics of diarrhea-related bacteria were discussed, which can be used as biomarkers. A total of 14 phylum types were identified from phylum to species, and LDA score > 4 was a high-dimensional biomarker (Figure 2E). It is noteworthy that the biomarkers of Dia_Ce group were Enterobacteriales, Bacteroides, unidentified_Enterobacteriaceae, and Escherichia_coli, while the biomarkers of Con_Ce group were Muribaculaceae, Ruminococcus_sp_Zagget7, and Barnesiellaceae.

### 3.3. The Transcriptome of Rabbits with Diarrhea

With optimization and quality control, a total of 234.069 GB high-quality readings were obtained. The average amount of data for each sample was about 6.75 GB, and the Q30 (%) of all samples was greater than 88%. The GC content in two groups was about 44~57% (Table S3), which met the requirements of transcriptome sequencing and further analysis and statistics, and in 36 sequencing samples, the number of reads that can be mapped to the genome accounted for more than 84% of all sequencing samples, indicating no pollution during sequencing (Table 3).

**Figure 3:**
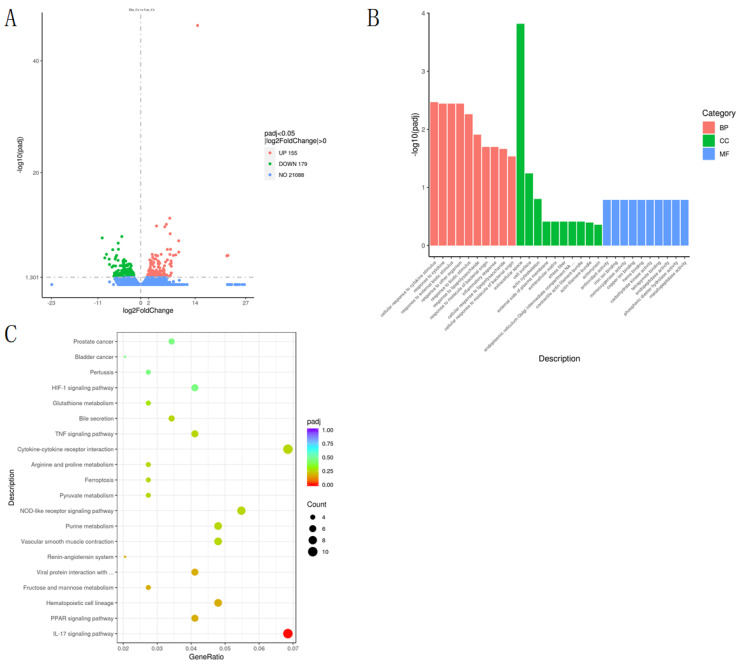
Differentially expressed genes in cecum of diarrhea-afflicted rabbits. (**A**) Volcano map of DEGs in cecum tissue of Con_Ce and Dia_Ce. (**B**) GO terms of Dia_Ce vs. Con_Ce. (**C**) KEGG pathways of Dia_Ce vs. Con_Ce.

A total of 334 differential expression genes (DEGs) were screened out in the Dia_Ce group (*p* < 0.05), including 155 upregulated genes and 179 downregulated genes (Figure 3A). In order to further study the functional distribution of 334 differentially expressed genes (DEGs), GO enrichment analysis results showed that a total of 2803 GO terms were mapped to differential expression genes (DEGs), of which 34 GO terms were mapped. The biological processes (BP) were mainly “cellular response to cytokine stimulus”, “response to cytokines”, “response to external biotic stimulus”, and “response to other organisms” (Figure 3B). In addition, DEGs were further analyzed by KEGG pathway, and the upregulated DEGs were mainly related to IL-17 signaling pathway, hematopoietic cell lineage, and viral protein interaction with cytokines and cytokine receptor (Figure 3C). Particularly, IL-17 signaling pathway and related genes MMP1, S100A9, and CXCL8 were significantly upregulated in Dia_Ce, and downregulated DEGs were mainly associated with vascular smooth muscle contraction and oxytocin signaling pathway.

### 3.4. Analysis of Differential Metabolites

**Figure 4:**
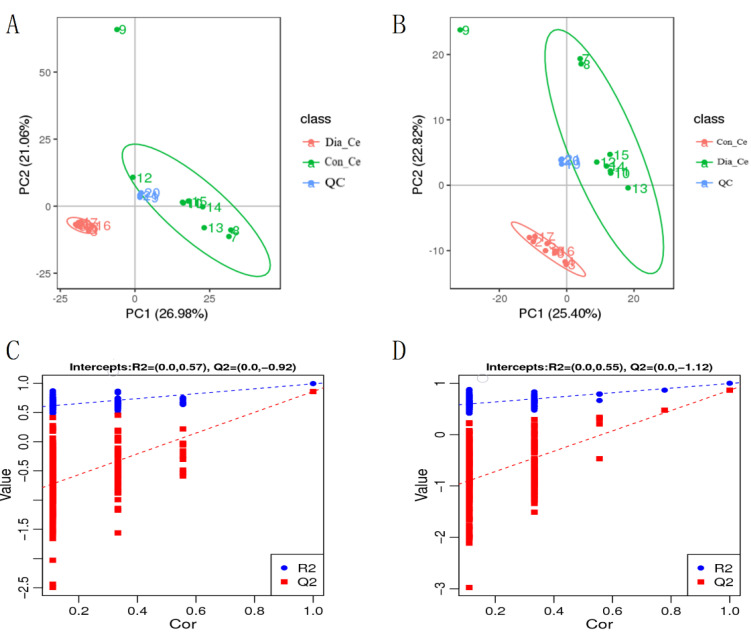
PCA and PLS-DA of fecal metabolites in cecum. (**A**,**B**) PCA score graph between Dia_Ce and Con_Ce; (**C**,**D**) permutation test from PLS-DA models. Note: (**A**,C): cationic mode; (**B**,**D**): anion mode.

The PCA analysis showed that metabolites were well separated into two groups (Figure 4A,B), and the QC samples were closely assembled in positive and negative ion modes, indicating that the repeatability of this experiment was good. In addition, the PLS-DA comparison diagram showed that there were significant differences between the two groups under positive and negative ion modes (Figure 4C,D).

**Figure 5:**
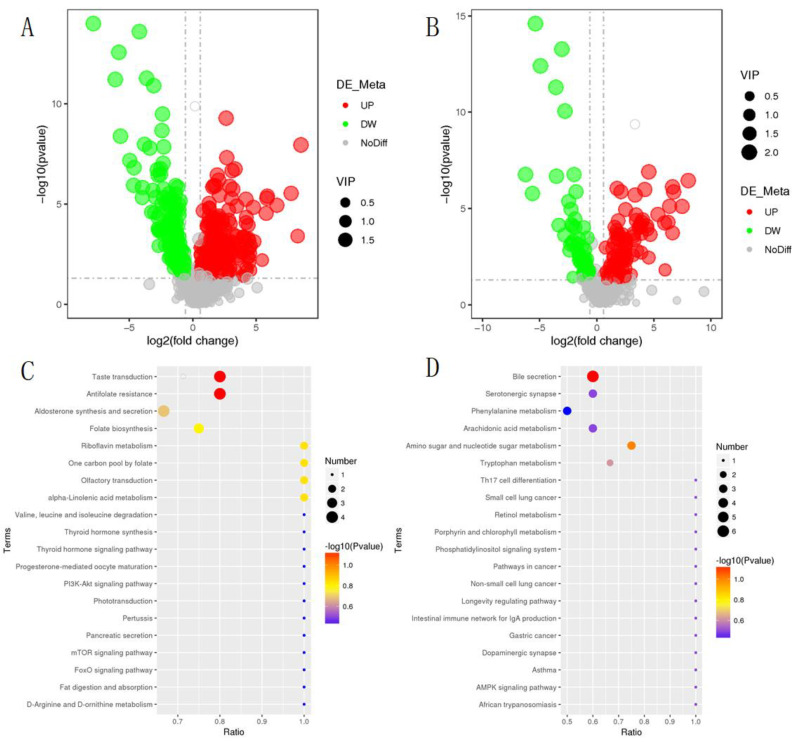
Differential metabolites and metabolic pathways in cecal feces of diarrhea-afflicted rabbits. (**A**,**B**) Volcano of metabolites with significant differences between Dia_Ce and Con_Ce, where A is a positive ion mode and B is a negative ion mode. (**C**,**D**) KEGG enrichment bubble diagram, where C is a positive ion mode and D is a negative ion mode.

Using endogenous substances with thresholds of VIP > 1.0, FC > 1.5, or FC < 0.667 and *p*-value < 0.05, a total of 652 metabolites were identified in Dia_Ce, among which 485 differential metabolites were identified in the positive ion mode, including 307 upregulation and 178 downregulation (Table 4). There were 167 differential metabolites in negative ion mode, including 102 upregulated and 65 downregulated (Figure 5A,B). The KEGG enrichment results are shown in Table 5. Bile secretion and amino sugar and nucleotide sugar metabolism were the most enriched pathways in Dia_Ce under positive ion mode (Figure 5C). In anion mode, the most enriched pathways were antifolate resistance, taste transduction, and alpha-linolenic acid metabolism (Figure 5D).

### 3.5. Correlation Analysis of DMs and the Cecum Bacteria and DEGs

**Figure 6:**
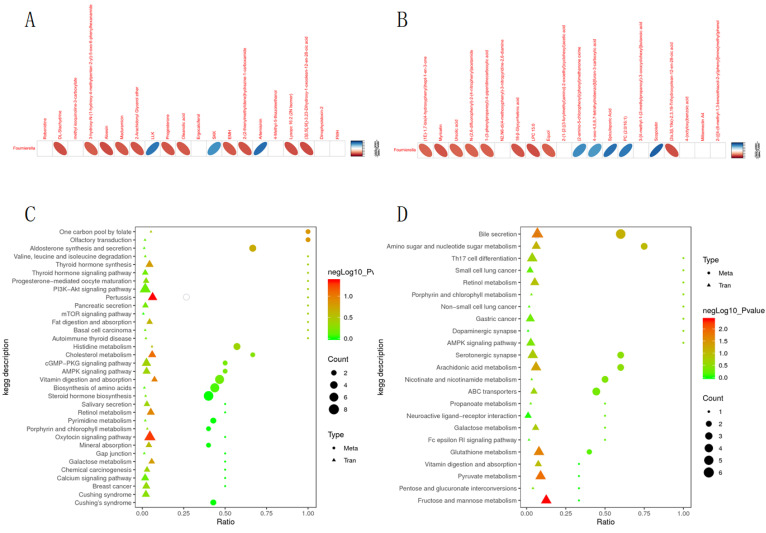
Correlation of DM with cecum bacteria and DEGs. (**A**,**B**) Correlation analysis results of metabolites top20 and 16S differential bacteria top10. (**C**,**D**) KEGG enrichment analysis of metabolism and transcription. Note: (**A**,**C**) are positive ion modes; (**B**,**D**) are negative ion modes.

In the positive and negative ion mode, a total of 20 metabolites (including myricetin, ursolic acid, etc.) were positively correlated with the changes of Fournierella, and eight metabolites ((phenyl) methanone oxime, 4−oxo−4,5,6,7−tetrahydrobenzo [38] furan−3−carboxylic acid, spiculisporic acid, etc.) were negatively correlated (Figure 6A,B). According to the KEGG pathway annotation information for different genes and metabolites, most differential metabolites and DEGs are enriched in PI3K-Akt signaling pathway, vitamin digestion and absorption, and bile secretion (Figure 6C,D).

## 4. Discussion

Diversity of gut microbes plays an important role in adaptation to environment and stable production performance of livestock and poultry [39,40]. In the study, 16S rRNA sequencing analysis showed that the alpha diversity index of microbial flora in the diarrhea group was significantly reduced, indicating that feeding with non-resistant diets reduced the disease resistance of rabbits [41,42], which is consistent with previous studies showing that the growth performance and immune performance of livestock depend on the stability of intestinal microbial flora structure [43,44]. The relative abundance of Proteobacteria was significantly different between the two groups. Antibiotics might reduce the contribution of Proteobacteria to host metabolism and digestion, while antibiotic-free diets may enhance its effect on host. Bacteroides and Firmicutes were involved in fermentation, and provide nutrition for the host [45,46]. Firmicutes and Bacteroidetes could promote energy metabolism and collection by increasing carbohydrate metabolism and improving lipid metabolism [47,48]. The relative abundances of Firmicutes and Bacteroidetes decreased in varying degrees, and the ratio of Firmicutes/Bacteroidetes showed a downward trend in the Dia_Ce group, indicating that the lack of antibiotics might reduce the intestinal ability to absorb nutrients and convert energy.

At the genus level, several dominant genera showed significant differences between the two groups, among which, Enterobacteriales was more abundant in the intestine of diarrheal rabbits. Previous studies showed that the abundance of Enterobacteriales was associated with low intestinal barrier function, which allows antigens from diet or bacteria to enter the circulation and activate the immune system [49]; it is speculated that Enterobacteriaceae might be involved in immune and metabolic defects in diarrhea.

The results showed that the relative abundance of Firmicutes in Con_Ce was significantly higher than that in Dia_Ce, and the LDA score was higher. Increased relative abundance of Firmicutes produced more short-chain fatty acids (SCFAs). Some studies showed that SCFAs might affect anti-inflammatory ability by regulating immune cell chemotaxis and reactive oxygen species release, inhibiting the production of proinflammatory cytokines TNF-α, IL1β, and nitric oxide, and the activity of NF-κB [50,51]. In short, the environment of the cecum of rabbit is complex, and its flora balance is easily damaged by stimulation. Diarrhea destroys the integrity of tight junction structure and physiological function between cecum epithelial cells, and increases cecum permeability, which reduces the abundance of beneficial bacteria and increases the probability of intestinal diseases.

Interestingly, most DEGs (including FABP6, S100A9, TNFAIP3, etc.) were enriched in stress and inflammatory responses by functional enrichment analysis. Downregulation of FABP6 expression occurred in Dia_Ce. As is known, FABP6 is part of the bile acid recovery system transported in the intestine. Intracellular bile acid binding protein FABP6 [52] and deletion of FABP6 gene in mice can lead to poor bile acid absorption [53], resulting in decreased immunity. S100A9 participates in innate immunity and mediates inflammatory response caused by infection [54]. TNFAIP3 (also known as A20) is a ubiquitin-modifying enzyme [55]. Some diseases related to TNFAIP3, such as inflammatory bowel diseases, involve the function of intestinal barrier [56,57,58]. Many studies have shown that TNFAIP3 is a protein related to intestinal diseases and can promote the function of intestinal barrier [59]. In the study, TNFAIP3 was abnormally expressed in the cecum tissues of diarrheal rabbits, indicating that diarrhea might cause damage to cecum barrier function and adversely affect autoimmunity in rabbits. In addition, some studies reported that IL-17 is a cytokine family (A–F) composed of six members, including inducing cytokines and chemokines involved in inflammatory reactions [60,61]. The abnormal expression of IL-17 family members would lead to autoimmune diseases and inflammation. In the study, CXCL8, CXCL5, CASP3, and other EDGs enriched in IL-17 signaling pathway were significantly upregulated, which might be related to the inflammatory and immune response of the cecum of diarrheal rabbits.

Due to the severe influence of intestinal microflora on intestinal metabolism, flora imbalance changes host metabolism [62]. Non-targeted metabolomics analysis of cecal feces further proved that diarrhea changed the metabolic function of the rabbits. There were significant differences in metabolites in two groups, which should be related to the different composition of cecum microorganisms. Some studies have found that myricetin has beneficial anticancer, antiviral, cell protection, treatment of cardiovascular disease, antioxidant, anti-inflammatory, and anti-obese effects [63,64,65]. In the study, myricetin was significantly downregulated in Dia_Ce, leading to the occurrence of cecum inflammation in rabbits, which was consistent with the results of previous transcriptome analysis. Bile acid could promote the digestion and absorption of lipids and vitamins in the intestinal tract, improve the immune function of the body, and maintain the health of the body [66,67]. BA regulates BA metabolism through binding to the related receptors, and it could be used as a key signal molecule to regulate glucose, lipids, and energy metabolism [68,69]. BAs might play a role in the enhancement of epithelial permeability associated with intestinal diseases [70], and rabbit intestinal inflammation is associated with bile acid levels [21].

Moreover, lithocholic acid is a kind of bile acid that protects intestinal epithelial barrier [71]. Studies [72] have found that the core of lithocholic acid AAA-10 is an effective tool to inhibit bile salt hydrolase activity and regulate bile acid pool composition in vivo. The use of anti-free diet reduced the content of lithocholic acid in the bile secretion metabolic pathway in the cecum of the rabbits, which might cause the metabolic disorders in the cecum of the host and reduce the immunity of the body.

The metabolic balance of the host is closely related to the stability of intestinal flora [73]. In the study, the changes of myricetin and ursolic acid were positively correlated with the level of Fournierella, and ursolic acid was enriched in tryptophan metabolism. Tryptophan metabolic disorder could cause intestinal microbial metabolic disorder and induce intestinal inflammation in rabbits [21,74]. In addition, Fournierella is a newly discovered genus in recent years that is sensitive to antibiotics such as amoxicillin, erythromycin, gentamicin, and penicillin G [75]. Combined with the previous description, we speculate that Fournierella might be involved in the bile secretion and the tryptophan metabolism in diarrhea-afflicted rabbits. In addition, we found that PI3K-Akt signaling pathway, vitamin digestion and absorption, and bile secretion had common pathways for the enrichment of most differential metabolites and DEGs.

## 5. Conclusions

The study found that the relative abundances of Firmicutes and Proteobacteria changed significantly in diarrhea-afflicted rabbits, and the differential metabolites were enriched and upregulated in bile secretion and tryptophan metabolism, which led to the decrease of nutrient absorption and energy-conversion ability in rabbits, and also caused the imbalance of intestinal mucosal immunity and inflammatory response and metabolic process. It is speculated that Fournierella might be one important reason for diarrhea in rabbits. Further research should be carried out to verify the function of DEGs in rabbit.

## Figures and Tables

**Table 1:** Alpha diversity of intestinal microbial of rabbits.

Group	Observed	Chao1	Shannon	Simpson	Coverage (%)
Dia_Ce	75.50	112.87	3.62	0.78	96.43
Con_Ce	336.33	1304.05	6.59	0.95	71.68
SEM	98.794	619.389	0.802	0.059	11,246
*p*-value	0.025	0.083	0.004	0.0163	0.052

**Table 2:** Cecum microflora taxonomic composition at order level.

Group	Clostridiales	Enterobacteriales	Bacillales	Bacteroidales	Verrucomicrobiales	Erysipelotrichales
Dia_Ce	0.205404	0.264739	0.005669	0.243197	0.134921	0.013228
Con_Ce	0.493008	0	0	0.256992	0.03099	0.00737
SEM	0.087	0.149	0.004	0.183	0.092	0.005
*p*-value	0.009	0.106	0.164	0.899	0.283	0.273

**Table 3:** Statistical table of comparison results between sample sequencing data and selected reference genome sequences.

Sample	Total Reads	Total Map	Unique Map	Multi Map	Positive Map	Negative Map
Dia_Ce_1	47079708	41,829,313 (88.85%)	36,717,519 (77.99%)	5,111,794 (10.86%)	18,315,048 (38.9%)	18,402,471 (39.09%)
Dia_Ce_2	40705516	36,216,676 (88.97%)	33,688,313 (82.76%)	2,528,363 (6.21%)	16,836,322 (41.36%)	16,851,991 (41.4%)
Dia_Ce_3	44406510	38,983,466 (87.79%)	36,626,529 (82.48%)	2,356,937 (5.31%)	18,271,015 (41.14%)	18,355,514 (41.34%)
Con_Ce_1	43584230	38,754,099 (88.92%)	34,649,374 (79.5%)	4,104,725 (9.42%)	17,294,670 (39.68%)	17,354,704 (39.82%)
Con_Ce_2	40285156	34,846,494 (86.5%)	31,066,590 (77.12%)	3,779,904 (9.38%)	15,488,047 (38.45%)	15,578,543 (38.67%)
Con_Ce_3	46651768	39,602,947 (84.89%)	37,114,380 (79.56%)	2,488,567 (5.33%)	18,502,785 (39.66%)	18,611,595 (39.89%)

**Table 4:** Screening results of metabolites differences.

Compared Samples	Num. of Total Ident.	Num. of Total Sig.	Num. of Sig. Up	Num. of Sig. Down
Dia_Ce. vs. Con_Ce. pos	1251	485	307	178
Dia_Ce. vs. Con_Ce. neg	493	167	102	65

**Table 5:** KEGG enrichment analysis of differential metabolites in Hyplus rabbit cecum feces samples from diarrheal rabbits (Dia_Ce) and healthy rabbits (Con_Ce) ^1^.

	Map ID	Map Title	*p*-Value	N	Meta IDs
ESI-	map04976	Bile secretion	0.07369	105	Salicylic acid, Reduced glutathione, Thromboxane B2, Lithocholic Acid, Chenodeoxycholic Acid, Deoxycholic acid
	map00520	Amino sugar and nucleotide sugar metabolism	0.098581	105	L-Fucose, N-Acetylneuraminic acid, N-Acetyl-α-D-glucosamine 1-phosphate
	map00380	Tryptophan metabolism	0.244399	105	Quinolinic acid, Picolinic acid
	map04152	AMPK signaling pathway	0.32381	105	NAD+
ESI+	map01523	Antifolate resistance	0.072914	182	Folic acid, Guanosine monophosphate, Adenosine 5′-monophosphate, 7,8-Dihydrofolate
	map04742	Taste transduction	0.072914	182	Serotonin, Guanosine monophosphate, D-Phenylalanine, Adenosine 5′-monophosphate
	map00592	alpha-Linolenic acid metabolism	0.146621	182	Jasmonic acid, Traumatic acid

^1^ ESI+: cationic mode; ESI-: Anion mode. Map ID = ID of enriched KEGG pathway; Map Title = Name of enriched KEGG pathway; and N = Number of backg.

## Data Availability

All the figures and tables used to support the results of this study are included.

## Supplementary Materials

The following supporting information can be downloaded at: The following are available online at https://www.mdpi.com/article/10.3390/ani12091194/s1, Table S1: Summary statistics of the 16S rRNA gene sequencing results. Table S2: Relative abundances of the top 11 components of the microbiota at the phylum level. Table S3: Summary statistics of the RNA sequencing results.
